# Supplementary material for: Neural Reconstruction Integrity: A Metric for Assessing the Connectivity Accuracy of Reconstructed Neural Networks
Source: Front Neuroinform. 2018 Nov 5;12:74. doi: 10.3389/fninf.2018.00074 (PMC6231021; doi:10.3389/fninf.2018.00074)
Supplement: Supplementary file 1 [file Data_Sheet_1.PDF]

Supplementary Material:
Neural Reconstruction Integrity: A metric for assessing the connectivity accuracy of reconstructed neural networks

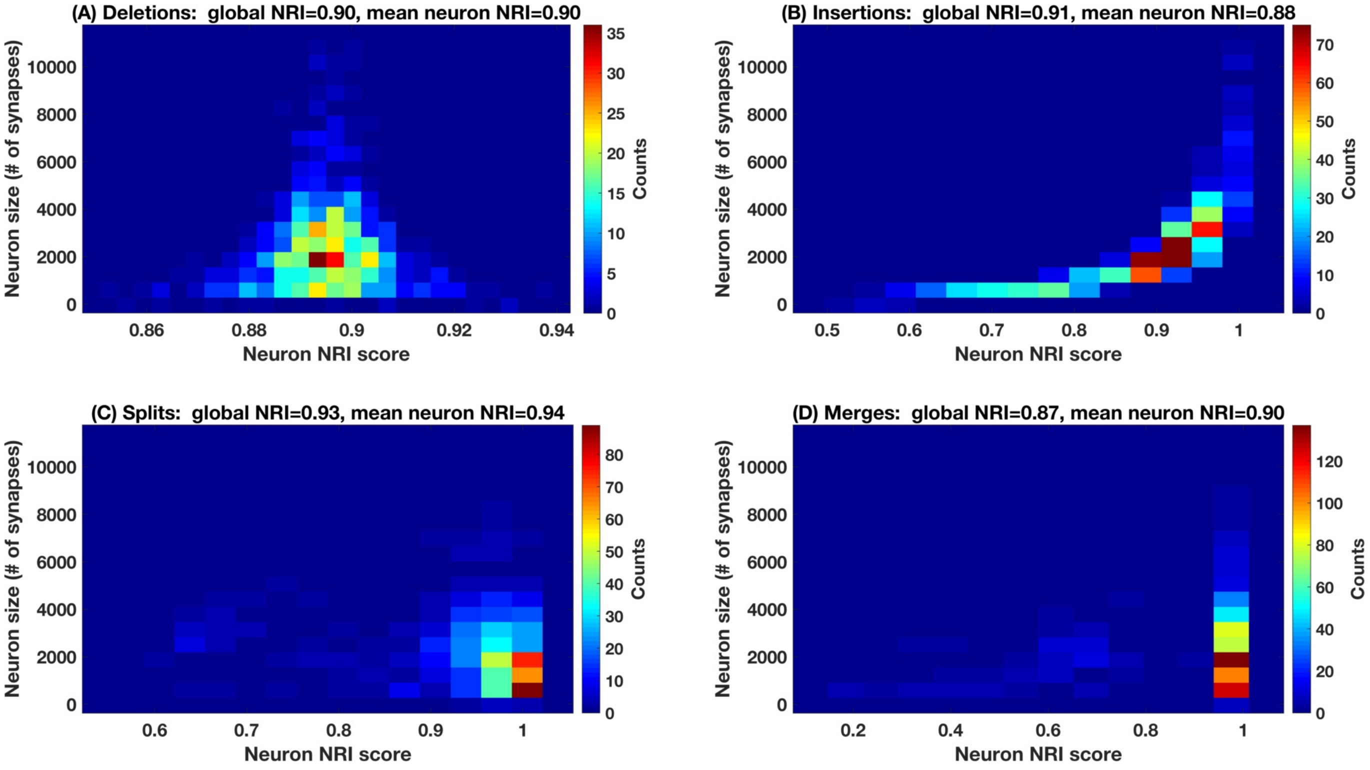

Figure S1. Neuron NRI scores from reconstructed networks of Figure 3 were paired with their respective neuron size (number of synapses) and plotted in 2-D histograms. Each histogram contains neurons from a single reconstruction network—that of Figure 3 which had a global network NRI score closest to 0.9 for the corresponding perturbation model. In all cases, global NRI scores are within about 3% of the average neuron NRI score. Under the tested conditions and perturbation models, large neurons do not appear to dominate influence on the global score. For example, the global score for the insertion error model (panel B) is close to the mode of the distribution, despite having a number of mid- and large-sized neurons with neuron NRI scores of 1.0. Distribution shapes generally reflect the perturbation models, with tight NRI distributions for models that introduce errors uniformly over neurons (e.g., deletions, which are independent of relative neuron locations) and broader distributions for models that consider more complex neuron relationships when introducing errors (e.g., insertions—which depend on the proximity between two neurons).
